# Successive grinding and sieving as a new tool to fractionate polyphenols and antioxidants of plants powders: Application to *Boscia senegalensis* seeds, *Dichrostachys glomerata* fruits, and *Hibiscus sabdariffa* calyx powders

**DOI:** 10.1002/fsn3.1022

**Published:** 2019-04-22

**Authors:** Markusse Deli, Elie Baudelaire Ndjantou, Josiane Thérèse Ngatchic Metsagang, Jeremy Petit, Nicolas Njintang Yanou, Joël Scher

**Affiliations:** ^1^ Food Sciences and Nutrition, ENSAI University of Ngaoundere Ngaoundere Cameroon; ^2^ Laboratoire d'Ingénierie des Biomolécules (LIBio) Université de Lorraine Vandœuvre‐lès‐Nancy France; ^3^ Department of Biological Sciences, Faculty of Sciences University of Ngaoundere Ngaoundere Cameroon

**Keywords:** antioxidant activity, chemical composition, particle size, plant powders, polyphenols, sieving

## Abstract

The present investigation aimed at evaluating the effect of powder fractionation based on particle size on the chemical composition in macronutrients, polyphenol contents, and antioxidant properties of powders of *Boscia senegalensis* seeds, *Dichrostachys glomerata* fruits, and *Hibiscus sabdariffa* calyces. Significant differences (*p* < 0.05) among granulometric classes of each plant were observed for the chemical composition in macronutrients. A decrease in particle size of plant powders was associated with an increase in ash, protein, and fat contents, while carbohydrate content was lowered. The following Granulometric classes, [0–180 µm] for *Boscia senegalensis*, [180–212 µm] for *Dichrostachys glomerata,* and [212–315 µm] for *Hibiscus sabdariffa,* respectively, were found to maximize total phenolic content and antioxidant activity. These results confirm that the grinding and controlled differential screening technology is an approach may serve as a useful guide to obtain optimum polyphenol extraction and enhance antioxidant activity of plant products*.*

## 1 INTRODUCTION

The interest for food supplements derived from medicinal plants has recently increased. This kind of natural supplements allows meeting the need for alternatives to conventional medicine, while surfing on the wave of “natural” or “organic” products. Plants are known to contain numerous bioactive molecules including terpenes, polyphenols, flavonoids, and alkaloids having various physicochemical properties and presenting a wide variety of biological activities, such as anti‐inflammatory, antimicrobial, and antioxidant activities (Da‐Costa‐Rocha, Bonnlaender, Sievers, Pischel, & Heinrich, 2014; Jafarian, Mortazavi, Kenari, & Rad, 2014; Kuate, Etoundi, Judith, Wan, & Julius, 2013). It is well established that plants constitute a significant source of bioactive molecules for the production of nutraceutics, functional foods, and additives (Joana Gil‐Chávez et al., 2013).

The use of plants in food supplements is based on traditional practices (powders, aqueous, or dry extracts) and modern extraction techniques designed for bioactive extraction from plants. However, the use of solvents for the extraction of active ingredients has currently brought up many criticisms (Palmade‐Le Dantec & Picot, 2010; Baudelaire, 2013): Worries have been reported in relation to the dangerousness of many solvents used for extraction (e.g., dichloromethane, acetyl acetate, and toluene), their impact on atmosphere, environment, and human health, the costs associated with the treatment of generated toxic wastes, and the impact on extracts quality and safety (Palmade‐Le Dantec & Picot, 2010).

Recently, alternation of drying and grinding process (ADG) and CDS extraction, that is, grinding and controlled differential screening, consisting in combinations of drying, grinding, and controlled sieving processes, has received increasing attention due to the raising desire to develop ecological extraction technologies of natural and active ingredients (Baudelaire, 2013; Becker et al., 2017,2016; Brewer, Kubola, Siriamornpun, Herald, & Shi, 2014; Karam, Petit, Zimmer, Djantou, & Scher, 2016; Li et al., 2015; Lucas‐González, Viuda‐Martos, Pérez‐Álvarez, & Fernández‐López, 2017; Zaiter, Becker, Baudelaire, & Dicko, 2018; Zaiter, Becker, Karam, & Dicko, 2016; Zaiter, Becker, Petit, et al., 2016). Indeed, the competitive advantage of plant powders in comparison with conventional bioactive molecules obtained from plants by solvent extraction mainly resides in the preservation of bioactive ingredients, and more especially of their bioactivity, for human interest. Becker et al. (2017) reported that the sieving process separates plant powders by granulometric differentiation through sieves of decreasing mesh, leading to selective distribution of bioactive molecules in the different granulometric fractions. Moreover, these authors explained that various parts of the same plant, more or less hard, more or less fibrous, are thus more or less difficult to crush, and thus, different plant parts may likely lead to particle fractions presenting different chemical compositions and/or structures. The sieving process leads to the separation of particles according to their sizes, thus leading to different physicochemical properties of resulting particle size classes (Guerrero‐Beltrán, Jiménez‐Munguía, Welti‐Chanes, & Barbosa‐Cánovas, 2009; Toth et al., 2005; Wang & Flores, 2000). The fine particles issued from the combination of drying, grinding, and sieving processes enable a better release of bioactive substances owing to their high specific surface area (Rosa, Barron, Gaiani, Dufour, & Micard, 2013; Zhao et al., 2018). Several recent studies have reported the link between particle size range of plant powders, bioactive ingredient contents, physicochemical properties, and functionalities (Becker et al., 2017; Sharma, Kadam, Chadha, Wilson, & Gupta, 2013; Zaiter, Becker, Karam, et al., 2016). With the increasing quantity and variety of powdered ingredients used in industry (Sharma et al., 2013), production of plant powders constitutes an important step for their valorization in various industrial sectors. Plant processing into powders not only allows the production of functionally adequate products, but also ensures their preservation during an extended shelf life, while supplying bioactive molecules under an adapted form for market of food supplements.

Africa is home to some of the most important species‐rich biodiversity regions in the world (Linder, 2014). In several African countries, natural products constitute an important part of human diet and are also an excellent source of bioactive molecules. First plant of medicinal interest of the current study, *Hibiscus sabdariffa,* is an edible plant, for which previous studies on alcoholic and aqueous extracts from its calyx reported anti‐inflammatory, antioxidant, hypolipidemic (Chung, Kong, Choi, & Kong, 2017; Medina‐Carrillo et al., 2015), and anti‐hypertensive effects (Abubakar, Ukwuani, & Mande, 2015), owing to its wealth of bioactive compounds (polyphenols, flavonoids, saponins, tannins, alkaloids, etc.). Then, it has also been showed that the aqueous, alcoholic, and hydroalcoholic extracts of the fruits of *Dichrostachys glomerata*, which is the second plant of medicinal interest of the study, exhibit antioxidant (Kuate, Etoundi, Soukontoua, Ngondi, & Oben, 2010), anti‐hypertensive, hypoglycemic (Fankam, Kuete, Voukeng, Kuiate, & Pages, 2011), anti‐inflammatory, and anti‐hyperlipidemic activities (Kuate et al., 2013); these numerous biological activities were attributed to their contents in a broad range of bioactive molecules such as alkaloids, saponins, tannins, mucilage, glucocapparins, and sterols. Likewise, the seed extracts of the third plant of medicinal interest of the current study, *Boscia senegalensis*, are rich in saponins, tannins, anthraquinone, alkaloids, and flavonoids; it has been shown that they have anti‐inflammatory, anti‐hyperglycemic, and antioxidant properties (Dongmo, Dogmo, & Njintang, 2017).

This work aims at evaluating the effect of grinding and sieving on chemical composition in macronutrients, phytochemical contents, and antioxidant activity of powders of *Boscia senegalensis* seeds, *Dichrostachys glomerata* fruits, and *Hibiscus sabdariffa* calyces. It intended to find the granulometric fractions presenting the highest antioxidant activity for their use in nutraceutical formulations.

## 2 MATERIALS AND METHODS

### 2.1 Plant material

Samples were collected in May 2015 from different localities: Sun‐dried red calyces of *Hibiscus sabdariffa* were purchased from local markets in the Adamawa region of Cameroon, sun‐dried fruits of *Dichrostachys glomerata* were bought in a market located in Yaounde (Central Region, Cameroon), and dry fruits of *Boscia senegalensis* were purchased from rural farmers in a local market of Oum‐Madjer (Batha state, Chad). The fruits of *Dichrostachys glomerata* and *Hibiscus sabdariffa* calyces were manually separated from inorganic materials, dirt, and dust particles before grinding. On other hand, the fruits of *Boscia senegalensis* were manually decorticated and the obtained seeds were taken into grinding.

### 2.2 Chemicals

Ascorbic acid, aluminum chloride, gallic acid, sodium hydroxide, Folin–Ciocalteu reagent, sodium carbonate, methanol, ethanol, potassium ferricyanide (K_3_Fe(CN)_6_), potassium ferrocyanide (K_4_Fe(CN)_6_), rutin, catechin, vanillin, sulfuric acid, 6‐hydroxy‐2,5,7,8‐tetramethylchlorman‐2‐carboxylic acid (trolox), α‐tocopherol, butylated hydroxytoluene (BHT), butylated hydroxyanisole (BHA), 2,2‐diphenyl‐1‐picrylhydrazyl (DPPH), 2,2'‐azino‐bis 3‐ethylbenzothiazoline‐6‐sulfonic acid (ABTS), trichloroacetic acid, and anhydrous ferric chloride were purchased from Sigma‐Aldrich (Saint‐Louis, USA).

### 2.3 Methods

#### 2.3.1 Plant grinding

An electric ultra‐centrifugal mill ZM 200 (Retsch, Haan, Germany) supplied with a sieve with 24‐tooth rotor of 99 mm diameter and sieve drilled with 1 mm trapezoid holes; was used to grind dry plant parts by impact and shearing size reduction principles. Approximately 1 kg of *B. senegalensis*, 1 kg of *D. glomerata,* and 1.2 kg of *H. sabdariffa* were ground by 50 g batches at 6,200 *g* rotor speed and ambient temperature of about 20°C. This rotor speed was chosen as a compromise between grinding efficiency and local temperature increase in plant parts during grinding, as the latter is known to be enhanced at high rotor speed and lead to bioactive compounds alteration (Karam et al., 2016; Zaiter, Becker, Petit, et al., 2016).

#### 2.3.2 Plant powder sieving

The sieving process is based on the separation of particles from a granular material by making them pass through several sieves of decreasing mesh size (315, 212, and 180 µm in the current study). Ground plants were sieved with the Analysette 3 Spartan apparatus (Fritsch, Idar‐Oberstein, Germany), operating by vertical vibration. Basically, 100 g ground plant sample was sieved in permanent vibratory mode at 0.5 mm amplitude for 10 min. The fraction of the powder retained on each sieve was recovered and weighed for the calculation of the mass fraction of each granulometric class. A sample of unsieved plant powder was kept for comparison purposes. Resulting plant powders were then put in sealed polyethylene plastic bags and stored at 10°C until analyses.

#### 2.3.3 Particle size distribution

Particle size distribution of each the powder classes and unsieved plant powders was determined by laser diffraction (Mastersizer 3000, Malvern Instruments France, Orsay, France) at ambient temperature, supplied with the Aero S dry dispersion unit that uses high‐pressure air to disperse particles. The obscuration level was set lower than 2% to avoid multiple scattering by adjusting dispersion conditions at 1.5 bar air pressure: 30% air pressure, 30% feed rate, 2.5 mm hopper length for powder samples of *Dichrostachys glomerata* and *Hibiscus sabdariffa*; 70% air pressure, 70% feed rate, 2.5 mm hopper length for *Boscia senegalensis* powder samples.

Particle sizes were expressed in terms of equivalent spherical diameters in volume. Characteristic sizes of the particle size distribution, D10, D50, and D90, were measured, where DX means that X % of the volume of particles has a diameter inferior to DX. Also, the span, a common parameter related to the width of particle size distribution, was evaluated as follows:

(1) Span=(D90-D10)/D50.

#### 2.3.4 Macronutrient composition

Moisture content was determined by drying 5 g plant powder in an oven at 103°C during 24 hr until reaching constant weight, according to AOAC method 925.10 (AOAC, 1990). Total ash content was determined by incinerating from 3 to 5 g plant powder sample in a furnace at 550°C for 6 hr, then weighing the residue after cooling to room temperature in a desiccator, following the AOAC method 920.87 (AOAC, 1990). The crude nitrogen content was obtained using the Kjeldahl method by the AOAC method 991.20 (AOAC, 1990), and the protein content was deduced from it with a conversion factor of 6.25 (AACC, 1999). In the procedure of fat content determination [34], 8 g of powder samples was added to 30 ml of chloroform/methanol (2/1 [v/v]) and mixed for 20 min. The mixture was filtered under dinitrogen, and the residue was re‐extracted in 20 ml of the same solvent and filtered. The extracts were then mixed and allowed to separate after the addition of 0.02 ml of NaCl solution at 0.7 g/100 ml. The fat was recovered by rotary evaporation at 40°C under liquid nitrogen (Rotavapor R‐144 Büchi, Flawil, Switzerland), and the fat contents were calculated by weight difference. Powder samples were hydrolyzed in 1.5 N sulfuric acid, and the available sugars were quantified by the phenol method (Dubois, Gilles, Hamilton, Ribers, & Smith, 1956).

#### 2.3.5 Determination of Phytochemical composition

##### Extraction of phenolic compounds

2 g plant powder was mixed with 20 ml methanol/water (70/30 [v/v]). The mixture was subjected to maceration by stirring at 300 rpm for 24 hr at room temperature (18 ± 2°C) and then filtered through with a Whatman filter paper (GE Healthcare companies, China) of 2–3 µm pore size. Thereafter, the supernatant was brought to 15 ml by addition of extraction solvent and stored at 4°C until analysis. This choice of extraction procedure (maceration under agitation) allowed shortening the process of extraction, thus minimizing the contact time of plant sample with solvent and better preserving the bioactivity of extracted molecules. Besides, the extraction at ambient temperature was also a compromise between extraction efficiency and limitation of thermal alteration of extracted biomolecules (Ćujíc et al., 2015).

##### Determination of total phenolic content

Total phenolic content of the hydromethanolic extract of plant powders was determined using the Folin–Ciocalteu method (Wafa, Amadou, Larbi, & Héla, 2014). Briefly, 20 µl of filtered extracts was diluted with 2,980 µl distilled water. Then, 500 µl of 10% (v/v) Folin–Ciocalteu reagent was added and the mixture was mixed. After 3 min, 400 µl of saturated solution of sodium carbonate Na_2_CO_3_ (20% [w/v]) was added. After stirring, the tubes were placed at room temperature for 60 min and absorbance was measured at 760 nm using a spectrophotometer (Shimadzu UV‐VIS 1605, Tokyo, Japan). A calibration curve (*R* ^2^ = 0.99) was prepared using standard solutions of gallic acid (40, 80, 120, 160, 200, 240, and 280 g/L). Thus, total phenolic content was expressed as milligram gallic acid equivalents per gram dry weight (mg GAE/g DW) of plant powder.

##### Determination of flavonoid content

The total content in flavonoid compounds of the different samples was measured following the method of Dewanto, Wu, Adom, and Liu (2002). In brief, 0.1 ml diluted and filtered hydromethanolic extract was added to 2.4 ml of distilled water and 0.15 ml of 5% Na_2_NO_2_ (w/v). After 6 min, 0.3 ml of 10% aluminum chloride (AlCl_3_·6H_2_O) (w/v) was added. The mixture was kept at room temperature for 5 min, and 1 ml NaOH (1 M) was added. Absorbance was then measured at 510 nm by UV/visible spectrophotometry against the extraction solvent as blank. A calibration curve (*R* ^2^ = 0.99) was prepared using 20, 40, 80, 100, 120, and 140 g/L rutin as standards. The results were expressed in milligrams rutin equivalents per gram of dry weight (mg RE/g DW).

##### Determination of condensed tannins content

The content in condensed tannins of plant powders was evaluated using concentrated sulfuric acid to depolymerize tannins, which allowed them to react with vanillin and produce red anthocyanidins that were detected at 510 nm by UV/visible spectrophotometry (Sun, Ricardo‐Da‐Silva, & Spranger, 2008). 0.05 µl of diluted and filtered hydromethanolic extract was mixed with 3 ml of 4% vanillin (w/v), and 1.5 ml concentrated sulfuric acid was added. The mixtures were stirred and then kept at room temperature for 30 min. The absorbance was measured at 500 nm by UV/visible spectrophotometry against the hydromethanolic solvent (70/30) as blank. A calibration curve (*R* ^2^ = 0.99) was prepared using standard solutions of catechin (100, 200, 300, 400, 500, and 600 µg/ml). The content in condensed tannins was expressed as milligrams catechin equivalents per gram dry weight (mg CE/g DW).

#### 2.3.6 Determination of antioxidant activity

##### DPPH radical scavenging activity assay

Antioxidant activity was first evaluated by the DPPH method (Zhang & Yasumori, 2004), in which the electron donating capacity of the extracts was measured by whitening of the purple‐colored solution of 1,1‐diphenyl‐2‐picrylhydrazyl (DPPH) cation radical. This assay is based on the ability of antioxidants to scavenge the DPPH cation radical. Briefly, 2 ml of 0.1 mM DPPH methanolic solution was added to 0.5 ml hydromethanolic extract of plant sample at different concentrations (0.025, 0.05, 0.1, 0.5, 1, 5, 10, 100 mg/ml). The mixture was thoroughly stirred and incubated in the dark for 1 hr at room temperature. After that, absorbance of the mixture was measured at 517 nm by UV/visible spectrophotometry. Lower absorbance of the reaction mixture indicated higher free radical scavenging activity. The scavenging activity was estimated based on the percentage of scavenged DPPH radical using the following:

(2) $$\text{Scavenging activity}\;\left(\%\right)=\left(\text{Control Absorbance}-\text{Sample Absorbance}\right)\times 100/\text{Control Absorbance}).$$

The antioxidant activity was expressed as the concentration required to cause 50% DPPH scavenging, referred as IC_50_(µg*/*ml). Ascorbic acid which was used as reference standard at the same concentrations as plant extracts showed an IC_50_ value of 14.33 ± 0.58 µg/ml.

##### ABTS cation radical scavenging activity assay

The ABTS radical cation scavenging activity was measured according to the method described by Re et al. (1999) with slight modifications. In brief, ABTS solution was generated as follows: 6.62 mg of potassium persulfate and 38.4 mg of ABTS reagent were weighed in a glass beaker, 10 ml distilled water was added, and then the mixture was perfectly mixed. This solution was kept away from light and let stand for 16 hr at room temperature to yield a blue–green‐colored solution containing the ABTS cation radical. Afterward, the ABTS^+•^ solution was diluted with absolute ethanol until reaching an absorbance of 0.70 ± 0.22 at 734 nm. Free radical scavenging activity was assessed by mixing 150 µl of each test sample at 0.025, 0.05, 0.1, 0.5, 1, and 5, 10, 100 mg/ml with 2 ml ABTS^+•^ radical solution. The decrease in absorbance was measured 1 min after mixing the solution. The scavenging activity was calculated according to Equation 2. The antioxidant activity was expressed as the concentration required to cause 50% ABTS inhibition, noted IC_50_(µg*/*ml). Ascorbic acid standard showed an IC_50_ value of 12.33 ± 0.58 µg/ml.

### 2.4 Statistical analysis

Results of chemical composition, phytochemical contents, and antioxidant activity were each subjected to analysis of variance (ANOVA) to determine the occurrence of statistically significant differences among them (*p* < 0.05). Duncan multiple range test was used to determine the degree of significance of the difference between two means. Statgraphics Centurion version 15.1.0.2 was used for this purpose. Analyses were done in triplicate, and the results were expressed as means ± *SD.* Pearson correlation coefficients were calculated to examine further correlations between bioactive compound contents and antioxidant activity. Principal components analysis (PCA) was used to show the correlations between analyzed variables and closeness between fractions of plant powders.

## 3 RESULTS AND DISCUSSION

### 3.1 Particle size distribution

**Figure 1:**
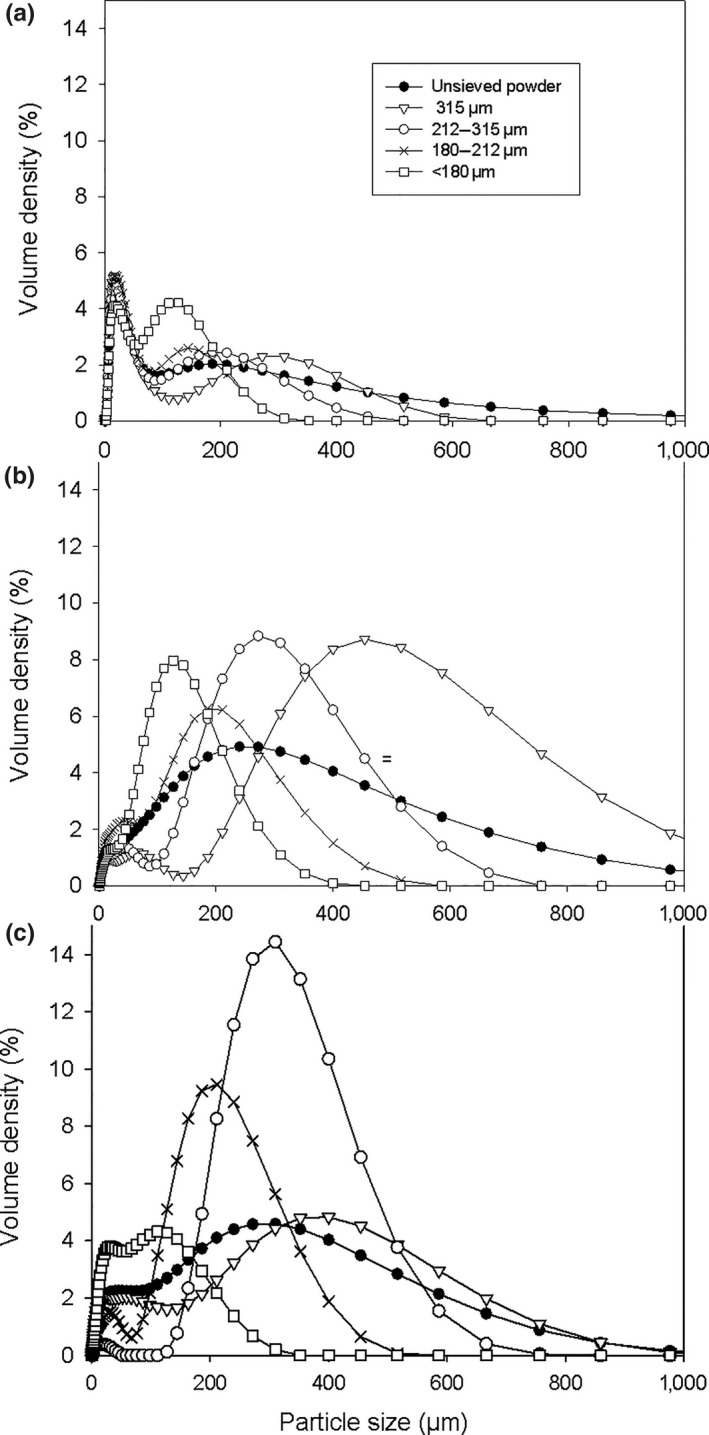
Particle size distributions of the different granulometric fractions and unsieved powder of *Boscia senegalensis* seeds (a)*, Dichrostachys glomerata* fruits (b), and *Hibiscus sabdariffa* calyces (c)*.* Full circles: unsieved powder; triangles: ≥315 µm; empty circles: 212–315 µm; crosses: 180–212 µm; empty squares: <180 µm

Particle size distributions of granulometric fractions and unsieved powders of *Boscia senegalensis* seeds*, Dichrostachys glomerata* fruits, and *Hibiscus sabdariffa* calyces are displayed in Figure 1. Unsieved powders of the three plants were bimodal, with the major population around 200 µm and the minor one around 20 µm for *Dichrostachys glomerata* and *Hibiscus sabdariffa*, and inversely for *Boscia senegalensis*. The lower particle size of *Boscia senegalensis* unsieved powder was also confirmed by its lower D50 of 28.60 ± 3.45 µm, compared to the D50 of *Dichrostachys glomerata* (166.00 ± 1.21 µm) and *Hibiscus sabdariffa* (126.00 ± 1.71 µm) unsieved powders.

In addition, the results of sieve fractionation and particle size characteristics of powder fractions (Table 1) showed that the grinding/sieving procedure was effective in separating *Dichrostachys glomerata* and *Hibiscus sabdariffa* powders into sufficiently different size classes. Indeed, D50 of granulometric fractions was comprised in or close to the sieve mesh range and span values were rather low (generally under 2), which is the sign of a relatively narrow particle size distribution and a quite homogeneous powder (Zhang, Xu, & Li, 2009). However, it is important to emphasize that during sieving analysis *Boscia senegalensis* was extremely sticky and cohesive, so it cannot be said that sieving analysis provided reliable results for *Boscia senegalensis* particle size. Sieving did not allow particles of *Boscia senegalensis* powder to be perfectly separated. Indeed, the high span values (over 4) of *Boscia senegalensis* fractions classes showed that they each were constituted of several particle size populations. Particle size distribution results of *Boscia senegalensis* powder samples were consistent with sieving issues encountered when processing this plant: *Boscia senegalensis* powder adhered to sieve walls and agglomerated upon vibrations, limiting its passage through sieve meshes, hence resulting in poorly efficient sieving difficulties.

**Table 1:** Sieved masses, mass fractions, and mean particle sizes of the different granulometric classes and unsieved powders of *Boscia senegalensis* seeds, *Dichrostachys glomerata* fruits, and *Hibiscus sabdariffa* calyces

Powder sample	Sieved masses (g)	Mass fractions (%)	Mean particle size *D*_50_ (µm)	Span (−)
*Boscia senegalensis*				
0–180 µm	149.5	15.06	35.53 ± 0.06^d^	4.25 ± 0.06^a^
180–212 µm	118.0	11.89	26.10 ± 0.30^b^	5.42 ± 0.12^b^
212–315 µm	356.0	35.86	26.63 ± 0.21^b^	7.13 ± 0.29^c^
≥315 µm	369.1	37.18	24.70 ± 0.10^a^	10.53 ± 0.01^e^
Unsieved powder	–	–	28.60 ± 1.01^c^	8.63 ± 0.36^d^
*Dichrostachys glomerata*				
0–180 µm	267.5	26.80	109.33 ± 0.58^a^	1.89 ± 0.02^a^
180–212 µm	178.4	17.86	115.33 ± 0.58^b^	2.54 ± 0.05^b^
212–315 µm	314.4	31.49	236.00 ± 2.65^d^	1.86 ± 0.04^a^
≥315 µm	245.1	22.55	397.33 ± 4.62^e^	1.87 ± 0.02^a^
Unsieved powder	–	–	166.33 ± 2.08^c^	3.09 ± 0.01^c^
*Hibiscus sabdariffa*				
0–180 µm	442.1	37.01	46.33 ± 0.51^a^	3.52 ± 0.05^c^
180–212 µm	171.2	14.33	174.33 ± 2.52^c^	1.72 ± 0.02^b^
212–315 µm	208.8	17.48	311.67 ± 1.15^d^	0.95 ± 0.02^a^
≥315 µm	173.3	14.51	504.50 ± 3.00^e^	0.91 ± 0.01^a^
Unsieved powder	–	–	125.67 ± 2.08^b^	3.72 ± 0.09^d^

Note

For each plant and granulometric parameter, means ± *SD* followed by the same superscripted letter were not significantly different (*p* < 0.05).

### 3.2 Biochemical composition

The proximate composition of fractions and unsieved powders is shown in Table 2. The moisture content of plants was ranging from 4.66% for *Boscia senegalensis* to 8.22% for *Hibiscus sabdariffa*, which makes them more stable during storage and packaging. Indeed, higher moisture content (generally over 10%) induces the development of microorganisms and product deterioration (Kaur, Kaushal, & Sandhu, 2011). No clear trend about the influence of particle size can be drawn from moisture results. A similar observation was reported by Becker et al. (2016): According to these authors, the heating effect of grinding, more pronounced for smaller particles and leading to moisture content decrease by heat‐induced evaporation, could be compensated by the higher specific surface of small particles, facilitating the absorption of surrounding air humidity.

**Table 2:** Proximate composition of the different fractions and unsieved powders of *Boscia senegalensis*, *Dichrostachys glomerata*, and *Hibiscus sabdariffa*

Contents (g/100 g DW)	Granulometric classes				Unsieved powder
	0–180 µm	180–212 µm	212–315 µm	≥315 µm	
*Boscia senegalensis*					
Moisture	5.77 ± 0.20^c^	4.89 ± 0.19^a^	4.71 ± 0.08^a^	5.33 ± 0.33^b^	4.66 ± 0.01^a^
Ash	3.63 ± 0.16^c^	3.50 ± 0.34^bc^	4.31 ± 0.21^d^	3.17 ± 0.01^b^	2.24 ± 0.13^a^
Crude proteins	33.16 ± 0.60^d^	31.38 ± 0.50^c^	30.12 ± 0.54^b^	28.92 ± 0.04^a^	31.72 ± 0.39^c^
Crude fat	7.63 ± 0.07^c^	7.19 ± 0.21^b^	7.13 ± 0.11^b^	6.57 ± 0.13^a^	7.15 ± 0.15^b^
Carbohydrates	50.47 ± 0.50a	52.92 ± 0.16^b^	54.73 ± 1.00^b^	57.09 ± 0.90^c^	53.67 ± 0.50^b^
*Dichrostachys glomerata*					
Moisture	6.17 ± 0.17^a^	7.33 ± 0.33^c^	6.50 ± 0.17^ab^	6.89 ± 0.19^b^	6.11 ± 0.19^a^
Ash	4.48 ± 0.46^c^	3.96 ± 0.01^ab^	4.17 ± 0.09^b^	3.58 ± 0.35^a^	6.04 ± 0.01^d^
Crude proteins	16.14 ± 0.11^b^	15.93 ± 0.40^b^	15.93 ± 0.30^b^	15.70 ± 0.56^b^	14.91 ± 0.16^a^
Crude fat	2.68 ± 0.19^a^	4.22 ± 0.28^c^	3.75 ± 0.25^b^	2.57 ± 0.18^a^	2.42 ± 0.18^a^
Carbohydrates	18.16 ± 0.6^a^	19.01 ± 0.66^a^	23.17 ± 0.67^b^	31.25 ± 0.99^c^	22.12 ± 0.41^b^
*Hibiscus sabdariffa*					
Moisture	7.00 ± 0.01^a^	8.11 ± 0.38^c^	7.42 ± 0.37^ab^	8.22 ± 0.19^c^	7.83 ± 0.50^bc^
Ash	11.47 ± 0.36^bc^	10.88 ± 0.05^ab^	10.44 ± 0.32^a^	11.62 ± 0.34^c^	11.03 ± 0.48^abc^
Crude proteins	6.93 ± 0.08^ab^	6.89 ± 0.12^ab^	7.08 ± 0.21^b^	6.74 ± 0.20^a^	7.00 ± 0.16^ab^
Crude fat	4.30 ± 0.26^d^	2.92 ± 0.19^c^	2.26 ± 0.12^b^	1.46 ± 0.12^a^	2.39 ± 0.19^b^
Carbohydrates	65.25 ± 1.65^a^	67.54 ± 0.42^b^	67.11 ± 0.66^b^	72.51 ± 0.92^d^	69.41 ± 0.70^c^

Note

For each plant, means ± *SD* followed by the same superscripted letter were not significantly different (*p* < 0.05).

The results also showed that the chemical composition depends on particle size. A significant difference (*p* < 0.05) was observed between the protein content of the different fractions of *Boscia senegalensis*: Proteins were more concentrated in finer particles (<180 µm). On the contrary, protein contents were similar or at least very close for the different powder fractions of the two other plants.

It is often observed that the smallest particles are richer in minerals, because fibrous plant parts, containing fewer minerals, are harder to grind, resulting in larger particles (Becker et al., 2017,2016; Zaiter, Becker, Karam, et al., 2016). Similar observations were made on *Eucalyptus grandis* powders. In this respect, fractions with smaller particle size were found to possess higher ash content and smaller fiber (hemicellulose and cellulose) content (Flávia, Edwil, & Fernando, 2016). Liketotal ashes, the highest lipid content was observed for the smallest particles, confirming the results of earlier studies acquired on powders issued from other plants (Becker et al., 2016; Zaiter, Becker, Karam, et al., 2016).

### 3.3 Polyphenol composition

Table 3 presents the total polyphenol, flavonoid, and tannin content of the various plant powders of the study. The contents in these phytochemicals were well different between powder samples of the three investigated plants. Obtained results made it possible to sort the plants by descending order according to their contents in total phenolic and flavonoid compounds, as well as in condensed tannins: *Dichrostachys glomerata > Hibiscus sabdariffa > Boscia senegalensis*.

**Table 3:** Contents in total phenols, flavonoids, and condensed tannins in hydromethanolic extracts of the different granulometric fractions and unsieved powders of *Boscia senegalensis* seeds, *Dichrostachys glomerata* fruits, and *Hibiscus sabdariffa* calyces

Contents	Granulometric classes				Unsieved powder
	0–180 µm	180–212 µm	212–315 µm	≥315 µm	
*Boscia senegalensis*					
Total phenols (mg GAE/g DW)	2.05 ± 0.03^c^	1.93 ± 0.02^b^	1.99 ± 0.01^bc^	1.99 ± 0.02^bc^	1.60 ± 0.04^a^
Flavonoids (mg RE/g DW)	0.36 ± 0.01^d^	0.24 ± 0.01^b^	0.30 ± 0.01^c^	0.19 ± 0.01^a^	0.24 ± 0.01^b^
Condensed tannins (mg CE/g DW)	1.82 ± 0.01^a^	2.68 ± 0.01^c^	3.23 ± 0.31^d^	1.88 ± 0.01^b^	1.75 ± 0.08^a^
*Dichrostachys glomerata*					
Total phenols (mg GAE/g DW)	97.8 ± 0.6^d^	109.8 ± 0.7^e^	79.2 ± 0.2^b^	59.9 ± 0.6^a^	83.5 ± 0.8^c^
Flavonoids (mg RE/g DW)	94.2 ± 0.5^e^	75.3 ± 0.2^d^	69.6 ± 0.4^c^	48.7 ± 0.3^a^	66.0 ± 0.4^b^
Condensed tannins (mg CE/g DW)	73.6 ± 0.6^d^	72.7 ± 0.3^d^	51.8 ± 1.5^c^	24.0 ± 1.2^a^	41.0 ± 0.4^b^
*Hibiscus sabdariffa*					
Total phenols (mg GAE/g DW)	42.2 ± 0.2^d^	34.4 ± 0.8^b^	52.0 ± 0.2^e^	39.9 ± 0.5^c^	31.3 ± 0.7^a^
Flavonoids (mg RE/g DW)	21.6 ± 0.2^a^	28.4 ± 0.2^c^	30.5 ± 0.2^d^	22.6 ± 0.3^a^	25.8 ± 0.2^b^
Condensed tannins (mg CE/g DW)	20.8 ± 0.2^d^	20.3 ± 0.3^d^	19.0 ± 0.1^c^	15.1 ± 0.4^a^	17.0 ± 0.1^b^

Notes

CE: catechin equivalents; DW: dry weight; GAE: gallic acid equivalents; RE: rutin equivalents.

For each plant, means ± *SD* followed by the same superscripted letter were not significantly different (*p* < 0.05).

It can be noted that the content in phenolic components considerably varied between unsieved plant powders and powder fractions of the same plant. The <180 µm granulometric fraction of *Boscia senegalensis* presented a higher total phenolic content. This can be related to the highest total protein and lipid contents of this particle size fraction. Indeed, large particles, richer in carbohydrates and probably in fibers (Oghbaei & Prakash, 2016), are expected to contain less bioactive compounds (Becker et al., 2016). For the two other plant powders, intermediate size class presented the highest content in polyphenols: [180–212 µm] for *Dichrostachys glomerata* and [212–315 µm] for *Hibiscus sabdariffa*. The lower polyphenol content of smaller particles may be explained by the extreme rise in temperature (up to 90°C) experienced by fine particles during the grinding operation (Hu, Chen, & Ni, 2012). This temperature level is sufficient to alter polyphenols and flavonoids, thus reducing the antioxidant capacity of resulting powder (Hu et al., 2012). Indeed, particles of smaller size have greater specific surface area, thus they expose more bioactive molecules on their surface, leading to a higher oxidation rate of antioxidant molecules. Becker et al. (2016) and Becker et al. (2017) also described a loss of polyphenols during the grinding of *Salix alba*, *Hypericum perforatum*, and *Achillea millefolium*, the loss being more pronounced for smaller particles.

The link between bioactive compound and total protein contents was investigated by calculating Pearson correlation coefficients (*R*). The correlation between polyphenol and flavonoid contents for all plant powders was highly significant (*R* = 0.97; *p* < 0.05), and a more anecdotic correlation was found between total polyphenols and total proteins (*R* = 0.51; *p* < 0.05). This indicates that flavonoids constitute the most important class of phenolic compounds for studied plants. Condensed tannins are polymers of flavonoids or derivative residues of flavonols that linked by carbon–carbon bonds. This explains the markedly positive correlations (*R* = 0.97; *p* < 0.05) denoted between condensed tannins and flavonoids, as well as between polyphenols and condensed tannins (*R* = 0.96; *p* < 0.05). Similar correlations were reported on extracts of *Hibiscus sabdariffa* (Hakimeh, Shahryar, & Majid, 2016) and some other plants such as *Halimium halimifolium* (Rebaya et al., 2014) and *Thymus kotschyanus* (Baharfar, Azimi, & Mohseni, 2015). Strong amounts of bioactive contents are responsible for their more significant antioxidant activity.

### 3.4 In vitro antioxidant activity

#### 3.4.1 DPPH radical scavenging activity

The results of DPPH radical scavenging activity presented in Table 4 were expressed in terms of concentration required to cause 50% DPPH inhibition (IC_50_). The lower the IC50 value, the more important the antioxidant activity of the studied plant powder. From the DPPH assay, the maximal antioxidant activity (lowest IC_50_) was recorded for the [180–212 µm] fraction of *Dichrostachys glomerata* powder. For *Hibiscus sabdariffa* and *Boscia senegalensis*, maximal antioxidant activities were observed for the [212–315 µm] and [0–180 µm] size classes, respectively.

**Table 4:** Antioxidant activity (expressed in terms of IC_50_) of the granulometric fractions and unsieved powders of *Boscia senegalensis* seeds, *Dichrostachys glomerata* fruits, and *Hibiscus sabdariffa* calyces according to ABTS and DPPH assays

Powder fractions	Antioxidant activity (IC_50_, µg/mL)					
	*Boscia senegalensis*		*Dichrostachys glomerata*		*Hibiscus sabdariffa*	
	DPPH	ABTS	DPPH	ABTS	DPPH	ABTS
0–180 µm	49,500 ± 1,320^a^	21,500 ± 1,500^a^	38.67 ± 1.15^b^	77.33 ± 2.52^b^	46.00 ± 3.46^a^	273.33 ± 14.47^b^
180–212 µm	69,000 ± 500^c^	24,900 ± 660^b^	30.33 ± 0.58^a^	52.00 ± 1.00^a^	75.33 ± 0.58^c^	257.67 ± 4.04^b^
212–315 µm	56,000 ± 1,000^b^	32,450 ± 550^c^	56.33 ± 0.58^d^	120.33 ± 2.52^c^	67.50 ± 3.91^b^	237.67 ± 3.51^a^
≥315 µm	90,830 ± 290^e^	51,930 ± 1,100^e^	59.00 ± 1.00^e^	174.00 ± 1.00^e^	79.00 ± 1.00^cd^	393.33 ± 12.58^d^
Unsieved powder	86,160 ± 1,040^d^	37,000 ± 500^d^	42.00 ± 1.00^c^	145.00 ± 1.00^d^	81.67 ± 3.06^e^	314.67 ± 9.02^c^

Note

For each plant and antioxidant activity test, means ± *SD* followed by the same superscripted letter were not significantly different (*p* < 0.05).

The highest antioxidant activity in plant extracts was observed for *Dichrostachys glomerata* fruit powders followed by *Hibiscus sabdariffa* calyx powders, whereas *Boscia senegalensis* samples had the lowest DPPH free radical scavenging activities. The IC_50_ values of *Dichrostachys glomerata* and *Boscia senegalensis* unsieved powder extracts were tremendously different (42 and 86,160 µg/ml, respectively). Compared to ascorbic acid used as antioxidant reference, the fractions of *Dichrostachys glomerata* were 2–4 times and 4–14 times less active against the DPPH and ABTS radicals, respectively. Similarly, the *Hibiscus sabdariffa* powders were 3–6 times and 19–31 times less active against DPPH and ABTS radicals, respectively. This suggests that phenolic compounds significantly contributed to the antioxidant activity of investigated plant powders. This is consistent with the fact that antioxidant activity of plant products is generally attributed to radical scavenging activity of phenolic compounds such as flavonoids, polyphenols, and tannins (Rahman & Moon, 2007). The antioxidant activity of phenolic compounds is mainly due to their redox properties, which can play an important role in adsorbing and neutralizing free radicals, quenching singlet and triplet oxygen, or decomposing peroxides (Hasan et al., 2008). The high contents in bioactive compounds of studied plant powders are likely responsible for their significant antioxidant activity.

#### 3.4.2 ABTS radical scavenging activity

Table 4 also displays the antioxidant activity (IC_50_), in terms of ABTS radical scavenging activity, of the different granulometric fractions and unsieved powders of *Boscia senegalensis, Dichrostachys glomerata*, and *Hibiscus sabdariffa*. For each plant separately, significant differences (*p* < 0.05) were denoted between the antioxidant activity of the different fraction and unsieved powders. The maximum antioxidant activity was found in [0–180 µm], [180–212 µm], and [212–315µm] fractions, respectively, for *Boscia senegalensis, Dichrostachys glomerata,* and *Hibiscus sabdariffa*. Antioxidant activity deduced by the ABTS experiment allowed sorting plants according to their antioxidant activity in the following decreasing order: *Dichrostachys glomerata* > *Hibiscus sabdariffa* > *Boscia senegalensis*.

### 3.5 Principal Component Analysis of physicochemical and phytochemical properties of studied plant powders

**Figure 2:**
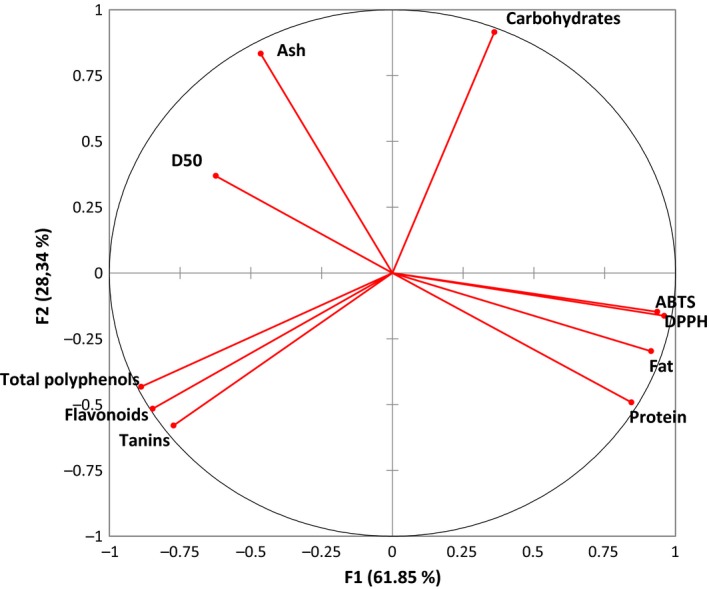
Representation of the variables of plant powders on the principal components F1 and F2

In order to compare all powder fractions of the three plants on the basis of their physicochemical, phytochemical, and antioxidant properties, measured parameters were organized into principal components by the principal component analysis (PCA) approach. The principal components 1 (PC1) and 2 (PC2), respectively, explained 61.9% and 28.3% variations among powder properties, allowing PCA to explain a total variation of 90.2%. Figure 2 gives the representation of analyzed powder parameters on the PC1 and PC2 axes. All powder properties highly contributed to the PC1xPC2 axes with the exception of D50 which contributed well to the PC3. The variables in PC1 that contribute much to discriminate the powders were DPPH and ABTS IC50, followed by fat, total phenol, flavonoid, and protein contents. Highly significant correlations (*r* > 0.95; *p* < 0.001) were found between phenols, flavonoids, and tannins, as attested by their proximity in the correlation circle of the PCA (Figure 2). Similarly, the concentration for 50% scavenging (IC50) of DPPH and ABTS (*r* = 0.98; *p* < 0.001) was almost superimposed in the correlation circle, as well as the fat and protein contents (*r* = 0.91; *p* < 0.001). We found negative linear correlation although lower (DPPH, *r *= −0.77; ABTS, *r* = −075; all *p* < 0.01), between the IC50 scavenging activities and the total phenol content. In addition, the D50 was significantly correlated negatively with protein (*r* = 0.61; *p* < 0.01) and fat (*r* = −0.76; *p* < 0.001) meaning that as the particle size increased, the protein and fat contents decreased. We can also observe in Figure 2 the opposition between the total carbohydrate and the phenol contents. In this respect, antioxidant activities of the powders were increased by the total phenol, flavonoid, and tannin contents while the protein and fat contents tended to alter them. These observations were in concordance with previous findings (Marhuenda et al., 2016; Singh, Singh, Ashish, & Salim, 2013).

**Figure 3:**
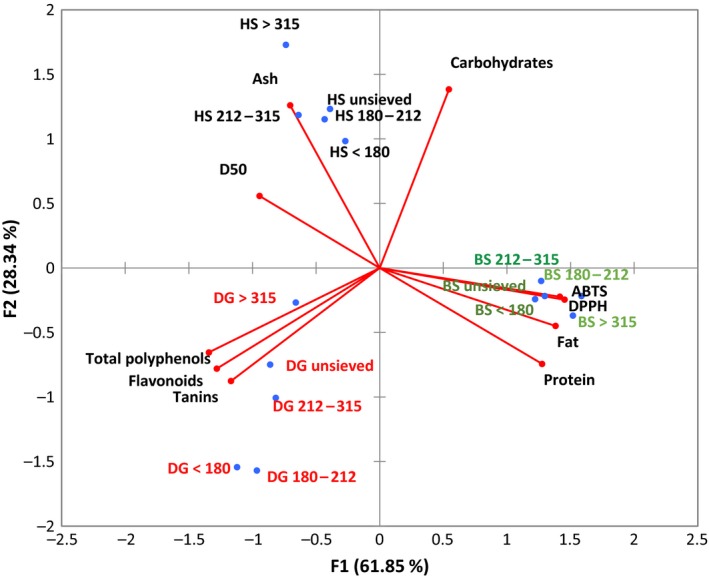
Representation of powder samples on the principal components F1 and F2. BS: *Boscia senegalensis*; DG: *Dichrostachys glomerata*; HS: *Hibiscus sabdariffa*

The representation of powder samples on the PC1 × PC2 plot revealed a separation of powder sample according to plant type (Figure 3). Generally, *Hibiscus sabdariffa* powders were positioned at the left frame‐up of the PC1xPC2 reference, characterized by their high content in ash and elevated mean particle size D50, along with low values of DPPH and ABTS IC50, fat, and protein contents; *Dichrostachys glomerata* powders were associated with high phenol, flavonoid, and tannin contents and relatively low levels of carbohydrates, while *Boscia senegalensis* powders were diametrically opposed to *Hibiscus sabdariffa*, as they were characterized by high protein and fat contents and great antioxidant activity, as well as low ash content. The figure also revealed relative high separation of the fraction powders of *Dichrostachys glomerata* and *Hibiscus sabdariffa*, while little separation was observed with *Boscia senegalensis* fractions. For both *Dichrostachys glomerata* and *Hibiscus sabdariffa,* the fraction powder position move from down to up as the size increased from [0–180 µm] to ≥350 µm with unsieved powder represented in between. This representation revealed the general tendency of increasing phenols content (more pronounced in *Dichrostachys glomerata* sample) as the particle size of the powder fraction decreased.

## 4 CONCLUSION

The aim of this study was to determine the granulometric class of *Boscia senegalensis* seed, *Dichrostachys glomerata* fruit, and *Hibiscus sabdariffa* calyx powders maximizing the antioxidant activity. It can be concluded that the compounds are distributed according to the particle size, particularly in the *Dichrostachys glomerata* and *Hibiscus sabdariffa* powders. Generally, smaller particles possessed higher contents of phenolic compounds and higher antioxidant activity than unsieved powders. Specifically, the highest polyphenol and flavonoid contents, along with the highest antioxidant activity, were found in the 0–180 µm, 180–212 µm, and 212–315 µm [granulometric classes for the powders of *Boscia senegalensis*, *Dichrostachys glomerata,* and *Hibiscus sabdariffa*, respectively]. This study shows the importance of the grinding and controlled differential screening technology in providing active and ecologic plant‐powdered extracts. Technology of grinding and sieving for improving phytochemical properties such as antioxidant activity and bioactive compound content of plants can be used in various applications such as foods ingredients, functional foods, and nutraceutics.

## CONFLICT OF INTEREST

The authors declare no conflict of interest.

## AUTHOR CONTRIBUTION

NYN, EBN, and JS have made substantial contributions to conception and design, while MD, TJNM, and JP contributed to acquisition, analysis, and interpretation of data, and drafting the manuscript. All the authors critically revised and approved the final submitted version of the manuscript. Prior to submitting the article, all authors agreed on the order in which their names are listed in the manuscript.

## ETHICAL STATEMENT

This study does not involve any human nor animal testing.
